# The central role of the gut in intensive care

**DOI:** 10.1186/s13054-022-04259-8

**Published:** 2022-12-07

**Authors:** Alberto Corriero, Raffaella Maria Gadaleta, Filomena Puntillo, Francesco Inchingolo, Antonio Moschetta, Nicola Brienza

**Affiliations:** 1 Department of Interdisciplinary Medicine - ICU Section, University of Bari “Aldo Moro”, Piazza Giulio Cesare 11, 70124 Bari, Italy; 2 Department of Interdisciplinary Medicine, University of Bari “Aldo Moro”, Piazza Giulio Cesare 11, 70124 Bari, Italy; 3 Dental Medicine Section, Department of Interdisciplinary Medicine, University of Bari “Aldo Moro”, 70124 Bari, Italy

**Keywords:** Microbiota, Microbiome, Intensive care, Dysbiosis, Probiotics, Prebiotics, Synbiotics, Fecal microbiota transplantation, Critical illness, Multidrug-resistant bacteria

## Abstract

Critically ill patients undergo early impairment of their gut microbiota (GM) due to routine antibiotic therapies and other environmental factors leading to intestinal dysbiosis. The GM establishes connections with the rest of the human body along several axes representing critical inter-organ crosstalks that, once disrupted, play a major role in the pathophysiology of numerous diseases and their complications. Key players in this communication are GM metabolites such as short-chain fatty acids and bile acids, neurotransmitters, hormones, interleukins, and toxins. Intensivists juggle at the crossroad of multiple connections between the intestine and the rest of the body. Harnessing the GM in ICU could improve the management of several challenges, such as infections, traumatic brain injury, heart failure, kidney injury, and liver dysfunction. The study of molecular pathways affected by the GM in different clinical conditions is still at an early stage, and evidence in critically ill patients is lacking. This review aims to describe dysbiosis in critical illness and provide intensivists with a perspective on the potential as adjuvant strategies (e.g., nutrition, probiotics, prebiotics and synbiotics supplementation, adsorbent charcoal, beta-lactamase, and fecal microbiota transplantation) to modulate the GM in ICU patients and attempt to restore eubiosis.

## Introduction

The total number of human cells is approximately 3.0·10^13^while the number of microorganisms inhabiting humans is approximately 3.8·10^13^ [1]. Collectively, they constitute the human microbiota, representing an organ itself [2].

**Fig. 1:**
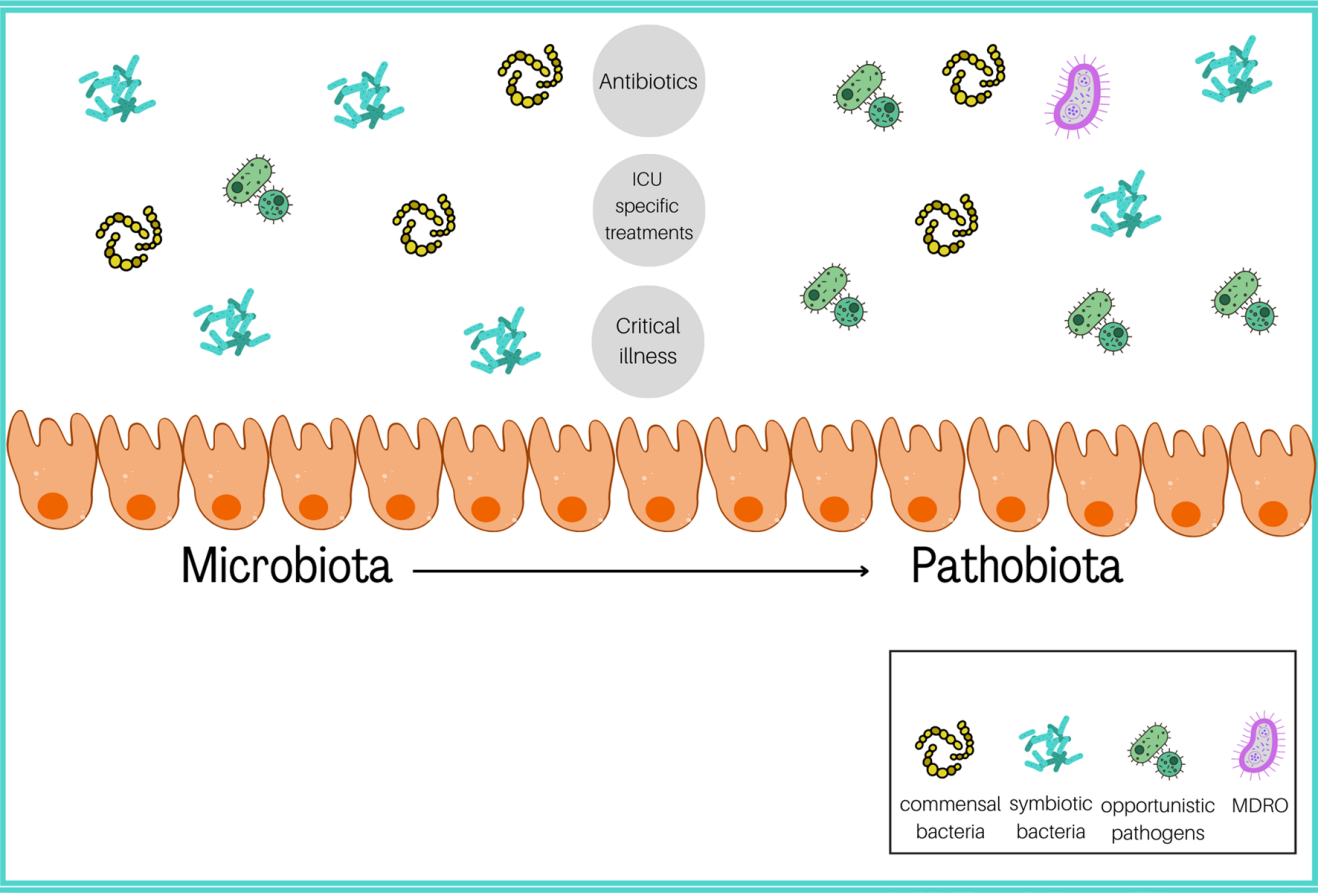
P1:The shift from microbiota to pathobiota in the ICU is driven by antibiotics and ICU-specific treatments like artificial feeding, mechanical ventilation, proton pump inhibitors, vasopressors, and opioids

Most of the microbiota colonizes the gut establishing a symbiosis with their host [3]. The gut microbiota (GM) of a healthy subject harbors all three main life domains: bacteria, archaea, and eukarya. The bacterial domain is the most represented [4]. There are six known bacterial phyla [5]. *Firmicutes* and *Bacteroidetes* are the most abundant, followed by *Actinobacteria* and *Proteobacteria* [6, 7]*.* The GM composition varies among individuals, changing throughout life due to intrinsic factors like age and genetics and extrinsic modifiable factors like diet [8], environment, and drug use [9]. In healthy individuals, the GM has prerogative functions, including enterocyte renewal modulation, metabolic and antimicrobic actions, and systemic activities such as improvement of glucose sensitivity [10], reduction of systemic inflammation, and even longevity [11]. Illness and drugs can disrupt the GM balance. In the intensive care unit (ICU), patients are subjected to antibiotics, gastrointestinal transit changes, nutritional changes, and sepsis [4], collectively leading to a GM imbalance, namely dysbiosis, whose most common symptom is diarrhea [12]. Ninety percent of the intestinal microflora is lost within 6 h of ICU admission [13]. ICU patients have lower bacterial diversity and variability, and opportunistic pathogens are enriched over symbiotics [14–16]. Most opportunistic pathogens are Gram-negative aerobic proteobacteria such as *Enterobacteriaceae* and Gram-positive bacteria such as *Staphylococcus *spp. and *Enterococcus *spp. [14]. This imbalance can lead to *Candida albicans* overgrowth in critically ill patients [2, 14, 15] while beneficial species such as *Ruminococcus *spp., *Pseudobutyrivibrio *spp., and *Faecalibacterium prausnitzii* become less represented [17, 18]. For these reasons, the GM in critically ill patients is defined as "pathobiota" (Fig. 1) [19]. The GM is also the main reservoir for "multidrug-resistant" bacteria (MDROs): Initially, these bacteria are counteracted by the resident beneficial bacteria, but then, as antibiotics eradicate them, MDROs take over and may promote infections during hospitalization [reviewed in [20]].

In critically ill patients, dysbiosis could be considered an organ dysfunction [21]. This review aims to describe this impairment and provide intensivists with a perspective on the currently available strategies to modulate the GM in ICU patients.

## The gut microbiota and the host

**Fig. 2:**
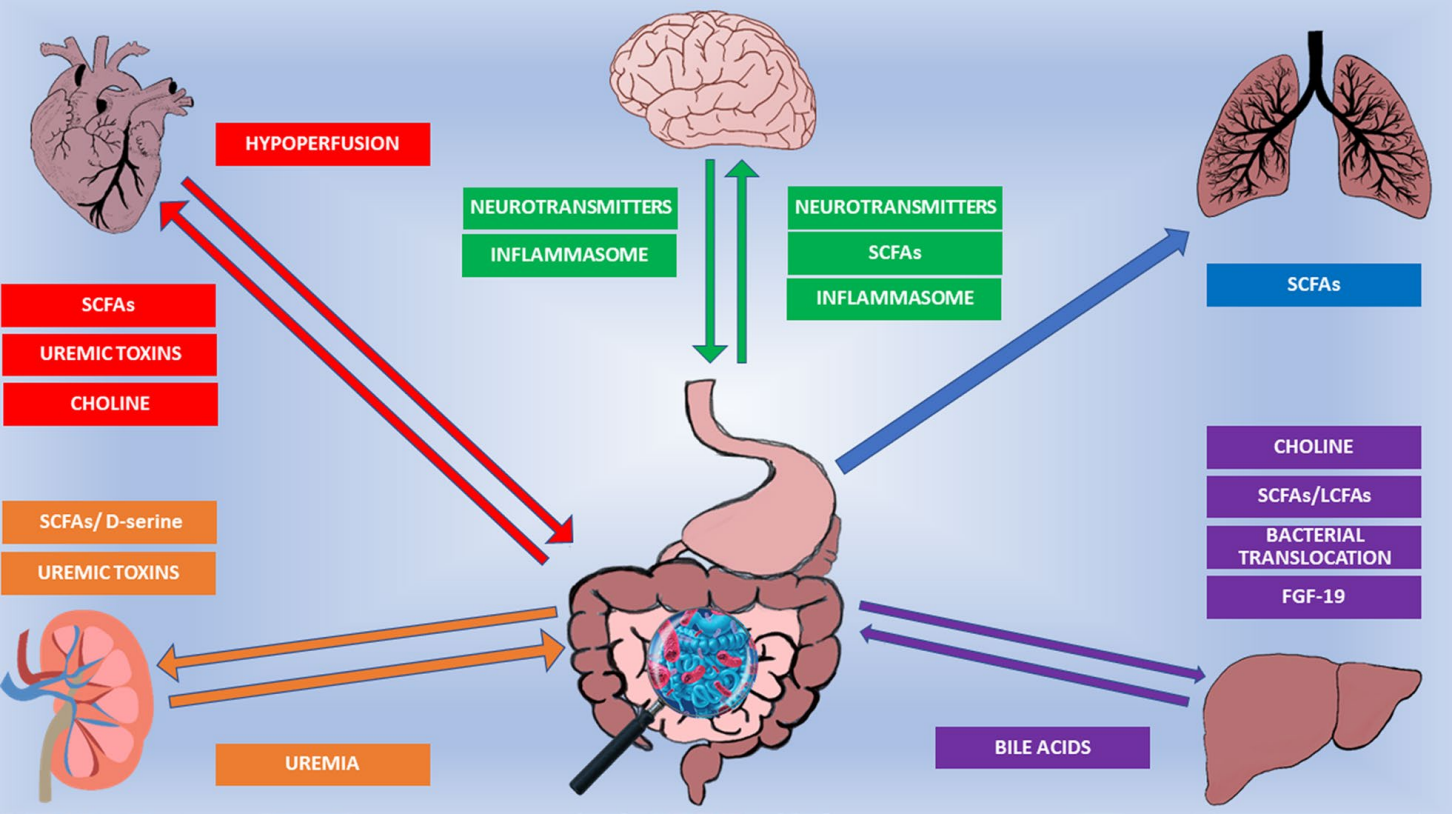
The GM interacts with its host along several axes. The GM is at the crossroad of multiple arrows representing molecular pathways involved in axes’communication. Further details in the main text. SCFAs: Short-Chain Fatty Acids. LCFAs: long chain fatty acids. FGF-19: Fibroblast growth factor 19

The GM interacts with its host (Fig. 2), and scientists are just beginning to characterize GM composition and function in health and disease. This paragraph describes the GM functions and how it interacts with the body, focusing on critically ill patients.

### The gut–brain axis

The blood–brain barrier (BBB) has always been considered molecule impermeable. However, immune cells, neurotransmitters, and some gut bacterial metabolites (SCFAs, vitamins, bile acids) can pass through it [reviewed in [22]]. These molecules affect memory, learning, behavior, and locomotion. Unbalanced molecular communication contributes to neurodegenerative and neuropsychiatric diseases [23], traumatic brain injury (TBI) [24], and sepsis-correlated brain impairment [25, 26]. The gut–brain axis (GBA) bidirectionally connects the central nervous system with the enteric one. It goes beyond a mere anatomical network and includes immune, endocrine, metabolic, and humoral communication routes. The GBA was first described in the early 2000s when antibiotic-treated germ-free (GF) or specific pathogen-free mice developed neurological problems [27]. The GBA links brain’s emotional and cognitive centers with the gut [28]. *Lactobacillus*, *Bifidobacteria*, *Enterococcus*, and *Streptococcus* produce acetylcholine,γ-aminobutyric acid (GABA), and serotonin [29]. Serotonin impacts brain functions, heart, bowel motility, bladder control, and platelet aggregation. Serotonin-based treatments in psychiatry and neurology regulate sleep, mood, and behavior [30]. Ninety-five percent of serotonin is produced from tryptophan in the gut by microbes [31, 32]. The GM also produces SCFAs butyrate, acetate, and propionate by fiber fermentation [22]. SCFAs contribute to maintaining the gut and BBB physiology. GF mice have increased gut and BBB permeability, and supplementation with the SCFAs-producing *Clostridium tyrobutyricum* restores both gut and brain homeostasis [33]. SCFAs influence the production of glutamate, glutamine, GABA, and neurotrophic factors. Propionate and butyrate modulate the expression of serotonin- and catecholamine-synthesizing enzymes and regulate intracellular potassium levels [reviewed in [34]].

Dysbiosis in the GBA is linked to neuroinflammation through the creation of the inflammasome, a biological complex of innate immune system multiprotein oligomers that activates inflammatory responses. Activated by pathogens or stress signals, the inflammasome assembles, producing pro-inflammatory cytokines [e.g.,interleukins (IL)-1 and IL-18] implicated in neuroimmunomodulation, neuroinflammation, neurodegeneration, and pyroptosis [35]. GM and inflammasome are strongly connected. Indeed, the inflammasome binds pathogen-associated and/or danger-associated molecular patterns, specific molecular motifs carried by gut microorganisms. Thus, a perturbation of the GM composition could overstimulate the inflammasome and compromise the GBA homeostasis, promoting neuroinflammation as seen in multiple sclerosis [36], Alzheimer's disease [37], Parkinson's disease [38], neuropsychiatric disorders [39], and sepsis [25, 26]. While no human studies are available, a recent preclinical study has shown how the GM plays a part in sepsis-associated encephalopathy by improving neurological outcomes when indole-3-propionic acid, a microbial neuroprotective metabolite, is produced, leading to the inhibition of NLRP3 inflammasome activation and IL-1β secretion in the microglia [26]. Similar preclinical results have been found in model of sepsis-induced cognitive decline [25], emphasizing the link between GM dysbiosis and the brain.

TBI is another condition compromising the GBA. Preliminary evidence on non-ICU patients shows that primary brain damage compromises the vagal nuclei and tractus solitarius nucleus [40], possibly leading to dysautonomia in the gastrointestinal tract and leaky-gut occurrence, which in turn affects BBB's permeability, reduces intestinal motility and leads to immune system dysregulation, as shown in a rat study [41].

### The gut–heart axis

The gut–heart axis is bidirectional. Beneficial microbial metabolites like SCFAs and uremic toxins (UT) wire this connection [42, 43]. Dysbiosis and, consequently, downregulated production of SCFAs concomitant to a high level of UT negatively affect the gut–heart axis. In this context, lower SCFAs and higher UT production have been observed. This is accompanied by increased absorption of lipopolysaccharide and endotoxin due to epithelial dysfunction, which ultimately triggers the systemic inflammatory response. This sequence of events facilitates the development of atherosclerosis and heart failure (HF) (reviewed in [44]). Conversely, it has been shown that HF causes dysbiosis, which promotes intestinal barrier damage, impairs nutrient absorption, and primes a vicious cycle leading to harmful microbial product translocation into systemic circulation, further aggravating HF [45–47]. Indoxyl sulfate (IS) and p-cresyl sulfate (PCS), UT derived from tryptophan and tyrosine fermentation, play a key role in this context. They promote fibrosis in klotho deficient and wild-type mice, negatively affecting heart and kidney function [48, 49]. Moreover, GF mice have less angiotensin II-induced hypertension and cardiac fibrosis than conventionally raised mice [50]. Observational data in patients with HF show decreased intestinal blood flow [46] and edema in the terminal ileum and colon [45], which lead to a change in the GM composition, with enrichment of *Campylobacter*, *Shigella*, and *Salmonella* [47]. Several HF therapeutic strategies targeting the GM, such as probiotic supplementation and diets, are being preclinically and clinically tested. In a rat ischemia–reperfusion model, probiotics administration reduces myocardial infarct size and remodeling [51].In a pilot human trial, *Saccharomyces boulardii* supplementation in HF patients improved left ventricular fraction and decreased left atrium diameter[52]. However, data are controversial so far, possibly because not all subjects included in these studies present with different grades of dysbiosis. The gutHeart trial studying GM manipulation to treat HF did not confirm the previous findings, possibly due to the lack of substantial dysbiosis in the enrolled patients[53].

A high-fiber diet prevents hypertension and cardiac hypertrophy in hypertensive mice by reshaping the intestinal microbial community GM and increasing the abundance of acetate-producing bacteria [54]. Similarly, a plant-based diet rich in complex carbohydrates and fibers while low in fat reduces HF events in both men [55] and women [56], improving arterial compliance, exercise endurance, and quality of life [57]. Choline is another soluble nutrient linked to HF. HF is worse in wild-type mice fed choline-supplemented diets [58]. Certain gut bacteria, such as *Enterobacteriaceae*[59]*,* can metabolize choline to trimethylamine (TMA). TMA should remain in the intestine; however, in a condition of leaky-gut secondary to other pathological conditions, TMA can translocate to the liver, where it is converted to trimethylamineN­oxide (TMAO), causing liver [60, 61] and heart damage [58]. Dysbiosis also promotes atherosclerosis. In fact, GF ApoE-deficient mice fed a low-cholesterol diet have more atherosclerosis plaques than conventionally raised mice [62]. However, when administered with *Akkermansia muciniphila*, which prevents endotoxemia-induced inflammation, they present with smaller atherosclerotic plaques [63].

As the emerging data show the contribution of the GM in the onset or progression of heart diseases and preclinical studies start to lay the foundation of interventions, data on the potential of these strategies in critically ill patients are lacking.

### The gut–lung axis

Evidence suggests that the lung and gut also communicate with each other, and complex pathways between their cognate microbiota strengthen the gut–lung axis (GLA). Neonatal gut dysbiosis with reduced *Bifidobacteria*, *Akkermansia*, and *Faecalibacteria* may cause CD4+ T cell dysfunction associated with childhood atopy and asthma [64]. IBD patients carry a higher risk of pulmonary diseases. In fact, IBD patients commonly have small and large airway dysfunction, obstructive and interstitial pulmonary diseases [65, 66] and, even when asymptomatic, may display early respiratory symptoms that could lead to bronchiectasis, mosaic perfusion, and air trapping, indicating obliterative bronchiolitis and centrilobular nodules and bronchial linear opacities bronchiolitis or bronchiectasis with mucoid secretion [67, 68]. Early after colectomy, several IBD patients developed respiratory symptoms. No bacterial pathogens were identified in these patients' sputum cultures, and corticosteroid therapy was required, suggesting a pulmonary impairment due to inflammation and not infection [69].

SCFAs may act as anti-inflammatory molecules and barrier keepers in the respiratory tract [70]. Mice administered with high-fiber diets display high intestinal SCFAs levels [71]. No SCFAs were found in lung tissue, but their levels promoted dendritic cell hematopoiesis and activated T helper 2 effector cells in the airways, establishing a de facto gut–bone marrow-lung axis protecting mice from allergic lung inflammation [71]. Wild-type mice depleted of GM with antibiotics, subsequently intranasally infected with pneumococcal pneumonia and then subjected to fecal microbiotal transplantation have an enhanced primary alveolar macrophage function thanks to a GM beneficial reshape [72].

The GLA could be a route for gut-to-lung bacterial migration. In a recent case–control study, *Klebsiella* and *Enterococcus* physically translocated to the bloodstream and pulmonary system, causing sepsis with disruption of GM diversity and enrichment of antibiotic-resistant bacteria, which led to secondary bloodstream and abdominal infections [73]. Acute respiratory distress syndrome(ARDS) could also be linked to GM dysbiosis as ARDS patients present enrichment of *Enterobacteriaceae* in the gut and lung while displaying lower bacterial diversity [74]*.*

### The gut–kidney axis

A dysbalanced GM has also been associated with kidney diseases, including acute kidney injury (AKI) [reviewed in [75]] and chronic kidney disease (CKD) [76]. UT, SCFAs, and D-serine establish the so-called gut–kidney axis. Abnormal levels of IS, PCS, and TMA contribute to the onset of renal tubular cell dysfunction, pruritus, fatigue, neurological damage, coagulation and endothelial dysfunction, mineral bone disorder, cardiovascular impairment, and insulin resistance, especially in patients affected by chronic kidney disease (CKD) [77]. Renal failure leads to higher urea concentration in both blood and intestine. This cause an overgrowth of intestinal bacteria owning urease activity, converting urea to ammonia [78], leading to dysbiosis. Patients with CKD and end-stage renal disease (ESRD) subjected to hemodialysis display enrichment of the genus *Faecalibacterium* and the families *Bifidobacteriaceae* and *Prevotellaceae*. Conversely, in ESRD patients subjected to peritoneal dialysis, the *Escherichia*genusand *Enterobacteriaceae*-*Enterococcaceae* families are predominant [79].UT also accumulate in AKI, but the underlying cause of this association remains uncovered [80].

Kidneys also express SCFAs receptors[81]. The olfactory receptor 78 (Olfr78) is a renal SCFA receptor, which promotes renin secretion. In experimental murine AKI models, butyrate decreases the production of reactive oxygen species and several pro-inflammatory cytokines [74] and exerts direct epigenetic activities by inhibiting histone deacetylases involved in the progression of glomerulopathies [82]. Renal T*oll-like receptor-4mRNA* levels are lower in mice supplemented with SCFAs, and inflammation is tampered [83]. Furthermore, *Bifidobacterium bifidum* BGN4 supplementation in mice previously subjected to bilateral renal ischemia–reperfusion injury attenuates AKI severity by modulating the inflammatory-immune response [84].

Free D-aminoacids are produced by bacteria in mice [85] and play a role in the physiology of several organs, including the kidneys. In fact, AKI-associated dysbiosis in mice leads to a lower D-serine associated with kidney injury aggravation [85]. Oral administration of D-serine alleviated renal damage pointing to D-serine’s renoprotective properties [85]. In the same study, authors have also shown a direct correlation between impaired kidney function in AKI patients and D-serine levels [83]. Despite the lack of mechanism of this association, these data suggest that D-serine levels could be a biomarker for AKI patients.

### The gut–liver axis

The portal vein, biliary tree, and systemic circulation connect the gut and liver. The liver interacts with the intestine by producing bile acids (BAs)[86], while the intestine interacts with the liver by metabolizing BAs, aminoacids, and exogenous components like alcohol and choline [87]. Dietary molecules, xenobiotics, free fatty acids, choline, and ethanol metabolites in the bloodstream reinforce this crosstalk [88]. Several signaling cues go through GM metabolism. In fact, gut dysbiosis contributes to several gut–liver conditions. *Enterococcus faecalis* contributes to alcoholic liver disease (ALD) [89], *Klebsiella pneumonia* to non-alcoholic steatohepatitis [90], and *Enterococcus gallinarum* to autoimmune hepatitis [91] pathogenesis.

BAs aid in the absorption of dietary fats and fat-soluble vitamins in the small intestine [92, 93] and act as signaling molecules by binding to the farnesoid X receptor (FXR) and G protein­coupled bile acid receptor 1 [94]. This triggers molecular events impacting their own hepatic synthesis(via the action of the FXR-FGF19 duo) and glucose and lipid homeostasis [94]. As BAs and GM communicate with each other, disrupting this delicate balance can lead to intestinal barrier impairment, inflammation, and even contribute to cancer onset and development. A damaged intestinal barrier allows bacteria to enter the portal vein and reach the liver, exacerbating liver [95] and intestinal diseases [96]. *Akkermansia muciniphila*, a gram-negative anaerobe colonizing intestinal mucus, is decreased in liver damage [97]. In this respect, bile duct ligated mice in which BA-FXR physiology is restored via the administration of a semisynthetic BA and FXR ligand, display a restored gut barrier integrity and *Akkermansia muciniphila* enrichment [96]. Loss of barrier caused by pathogenic bacteria disrupting epithelial integrity can lead to sepsis by triggering systemic inflammation (reviewed in [98]), and, in turn, sepsis increases the risk of liver damage [99]. Intriguingly, these studies suggest that sepsis-induced liver damage could potentially be prevented by harnessing the GM in ICU septic patients.

SCFAs and LCFAs are gut–liver messengers. Studies in rodents have shown that reducing SCFAs increases intestinal permeability [100] while reducing LCFAs decreases luminal *Lactobacilli* [101]. Supplementation with *Lactobacillus *spp. probiotics in animal models improves intestinal barrier integrity, inhibits colonization of infectious bacteria like *Clostridium difficile* and *Clostridium perfringens*, *Campylobacter jejuni*, *Salmonella Enteritidis*, *Escherichia coli, Staphylococcus aureus*, and *Yersinia*, and eases intestinal inflammation [102, 103].

Choline is metabolized into lecithin, contributing to VLDL hepatic excretion. Low choline may cause hepatic steatosis [104–106]. Lecithin levels depend on the host's choline-to-lecithin conversion. Any impairment in enterocyte metabolism could harm the liver by reducing lecithin supply. Personalized nutrition could slow the choline-TMAO pathway to prevent liver damage [60, 61, 107, 108].

Acute (caused by toxins or infections without underlying liver disease) or chronic (caused by pre-existing liver disease) liver dysfunction is common in ICU [reviewed in [109]]. No study has explored preventing or treating liver dysfunction in the ICU using GM modulators. Previous research on the adjuvant benefits of probiotics/prebiotics/symbiotics, fecal microbiota transplantation (FMT), and bacteriophages in non-alcoholic fatty liver disease [110, 111] and ALD [89, 112] could lay the groundwork for their future employment in other clinical contexts.

## Harnessing the microbiota in critically ill patients

**Fig. 3:**
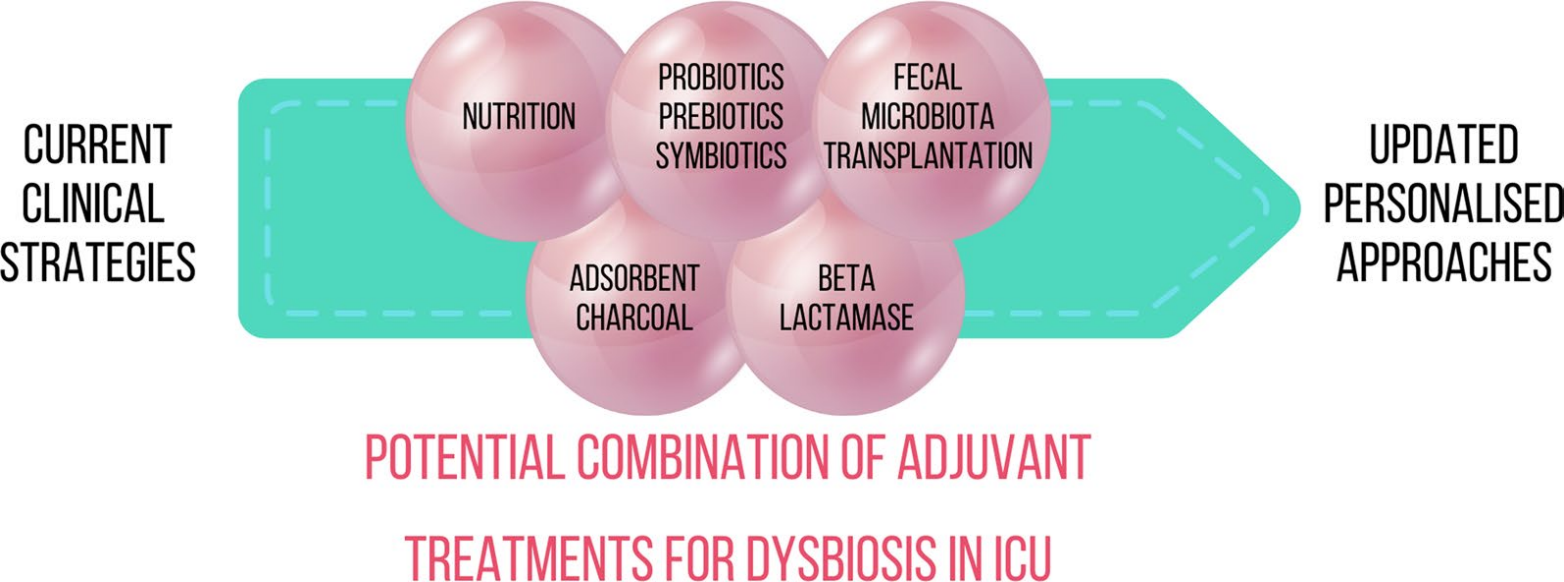
Potential personalized approach for treating dysbiosis in ICU patients

Despite the emerging data, there is a lack of human studies, especially on critically ill patients, and the available data should be expanded and validated in clinical trials. Moreover, a further effort to address biomarker discovery should be made in this field, as indicated by the potential shown by TMAO and D-serine. GM manipulation could be used as an adjuvant strategy in some comorbidities rising in critically ill patients. An overview of the current preclinical and clinical trials with grade of evidence with potential application to the ICU is provided in Table 1. Moreover, we propose a potential strategy (Fig. 3), where nutrition is the first and easiest step in managing dysbiosis in ICU patients, followed by probiotics, prebiotics, synbiotics, beta-lactamase, absorbent charcoal administration, and, finally, FMT. These treatments require validation with a high level of evidence before they can be routinely applied in ICU. They must be used with extreme caution aiming for a tailored approach to the patient with ideal efficacy and safety.

**Table 1:** Overview of the current preclinical and clinical trials with grade of evidence with potential application to the ICU

Interventions	Potential ICU applications	Grade of evidence
Nutritional approach (EN)	Promote GM eubiosis Reduce mortality/morbidity Reduce infectious complications	Very weak [113]
Adsorbent Charcoal	Prevention of RCDI	Very weak Pre clinical studies [114] One phase 2 trial completed[115]
Probiotics Prebiotics Synbiotics	Reduce infection rate, notably VAP	Weak [116–118] RCTs in ICU with low quality of evidence
	Coadjuvant in sepsis	Very weak [119, 120] Preclinical studies
	Coadjuvant in SARS-CoV-2 infection	Weak Ongoing RCTs in ICU [121–126]
	Coadjuvant in TBI	Very weak [127] Preclinical studies
	Prevention of RCDI	Very weak [128]
Beta-Lactamase	Prevention of RCDI	Very weak Phase 2 trials completed [129] One phase 1b/2a trial ongoing [130]
FMT	Treatment of RCDI	Weak [131, 132] One RCT [133] Four ICU retrospective cohort studies [134–137]
	Coadjuvant in TBI	Very weak [138] Pre clinical studies
	Coadjuvant in sepsis	Very weak [139, 140] Case reports and pre clinical studies

Evidence reported in ICU or critically ill settings have been specified

*ICU* intensive care unit, *VAP* ventilator associated pneumonia, *TBI* traumatic brain injury, *RCDI* recurrent *clostridioides difficile* infection, *FMT* fecal microbiota transplantation

### Nutrition

Different dietary patterns can modulate the GM in different ways. Quantity, quality, fiber content, and feeding patterns [141, 142] affect GM abundance and diversity [143]. ICU patients may undergo fasting or limited nutrition [144], resulting in dysbiosis [145]and compromised bacterial metabolite levels [4]. Nutrition in critical illness is a complex topic, and evidence is limited. Nevertheless, it has been observed that enteral nutrition (EN) benefits the GM more than parenteral nutrition (PN). PN raises *Bacteroidetes* levels and intestinal permeability in wild-type mice [146], while these effects are absent with EN [146]. GM is affected by the macronutrient-to-fiber ratio in EN formulas. A high protein and animal fat load will enrich GM *Bacteroides,* while carbohydrate-rich diets increase *Prevotella* strains [147]. Low fiber intake impacts gut epithelium integrity, mucus layer thickness, and the enrichment of pathogenic strains [148]. EN should be preferred over PN to preserve intestinal barrier integrity, prevent villi atrophy, and promote eubiosis, ultimately leading to lower mortality and morbidity [149] and fewer infectious complications [reviewed in [113]]. Anyhow, caution should be taken in formula selection. It has been observed that mice fed dietary emulsifiers show an imbalanced GM linked to colitis and metabolic syndrome [150]. Since EN formulas include preservatives and emulsifiers, like soy lecithin and glycerol derivates [151], they could potentially harm ICU patients.

### Probiotics, prebiotics, synbiotics

#### Probiotics

Probiotics are live microorganisms that provide a health benefit to the host when supplemented in sufficient quantities [152]. Probiotics promote eubiosis, reduce gut cell apoptosis, and support the immune system [153–155].

A meta-analysis analyzing several trials in about 2.900 critically ill patients on probiotic use in the ICU reported that strains such as *Saccharomyces boulardii*, *Lactobacillus *spp., and *Bifobacterium *spp. are associated with a reduction in infections, particularly in patients with ventilator-associated pneumonia (VAP) and treated with antibiotics, but not to increased survival [118]. Conversely, a more recent study did not confirm the beneficial role of *Lactobacillus rhamnosus GG* in reducing VAP incidence in ICU patients [156]. Another meta-analysis, which analyzed 4893 patients, has shown that probiotics reduce VAP, ICU length of stay, and duration of antibiotic therapy; however, the high variability in treatments and type of patients prevents the introduction of the use of probiotics as VAP prophylaxis [116]. Other positive effects of probiotics in specific patient subgroups, such as polytrauma patients, include improvement in clinical conditions, less use of vasopressors, reduction in the "sequential organ failure assessment" (SOFA) score, and a shorter ICU stay [157]. In particular, four probiotic preparations in polytrauma patients under mechanical ventilation lowered the incidence of VAP from 23.8% in the placebo group to 11.9% in the interventional group [157].

Three more meta-analyses analyzing the use of probiotics in critically ill patients to prevent VAP or mortality and ICU-acquired infections were recently published [117, 158, 159]. Consistently, authors concluded that probiotics administration is safe and beneficial and leads to a decrease in the incidence of ICU infections, notably VAP, whose prevention was the most effective in trauma patients. Conversely, the largest network meta-analysis, including 8339 patients from 31 RCTs, advocates that the safety of probiotics should be further studied, especially in critically ill patients, as in some cases, high dosages have been linked to an increase in infection complications such as sepsis, pneumonia, abscesses, and endocarditis due to bacteremia and fungemia [117].

Other potential indications for probiotics in ICU include sepsis, TBI, and, most recently, SARS-CoV-2 infection. Since 2007, the gut has been conceptualized as a "motor" that, when impaired, could drive systemic inflammation and multiple organ failure [160]. Experimental evidence point to *Faecalibacterium prausnitzii* as a probiotic strain potentially blunting systemic inflammation during sepsis as it produces an anti-inflammatory peptide that can counteract chemically induced colitis in mice [119]. Moreover, daily oral intake of *Lactobacillus rhamnosus GG and Bifidobacterium longum* has been shown to reduce mortality and improve intestinal epithelial homeostasis in a murine model of septic peritonitis [120]. Probiotics could also be a valid asset in TBI, as shown in mice subjected to traumatic spinal cord injury and supplemented with VSL#3, a mixture of eight bacterial strains [161]. This intervention supported mice's immune response in the gut and better locomotor recovery, improving post-injury outcomes [127].

The SARS-CoV-2 pandemic has impacted every field of medicine, including intensive care, where each virus variant has differently affected critically ill patients [162]. The role of probiotics in infected patients in intensive wards has not been systematically explored. However, their supplementation could influence the host's immune response [163]. Clinical trials studying the effect of probiotic supplementation in SARS-CoV-2 patients are ongoing or have recently been completed [121–126]. Emerging evidence points to the modulation of the immune function and reduction of secondary infections achieved by probiotics supplementation [164].

Next-generation probiotics are in development. SER-109 is obtained by transplanting fecal microbiota with alcohol-triggered massive sporulation. This new probiotic reduces the risk of recurrent *Clostridium Difficile* infection (RCDI) in patients treated with antibiotics per guidelines [128].

### Prebiotics and synbiotics

Prebiotics are specific nutrients for intestinal bacteria, while synbiotics combine prebiotics and probiotics. They can both be administered to modulate the GM [2]. A meta-analysis has shown that there is no difference in the incidence of infections in ICU patients between using probiotics alone or synbiotics [118]. Conversely, in VAP, synbiotics supplementation seems to produce a more significant benefit, like reducing infection rates, than probiotic supplementation alone [116, 117].

Despite the emerging evidence on harnessing the GM through the use of nutrition, pro-, pre-, and symbiotics, the fine mechanisms describing the published observation are still lacking, and this complicates the translation from bench to bedside. Moreover, caution should be taken. Studies on safety are imperative as these strategies do not come without pitfalls, as demonstrated by a recent retrospective study showing a correlation between probiotic administration and an increase in probiotic-associated central line infections leading to increased mortality [165].

### Beta-lactamase

GM changes in ICU patients are mostly due to broad-spectrum antibiotics, but not all induce dysbiosis [2]. Antibiotic stewardship includes switching from broad-spectrum molecules to a narrower spectrum and shortening antibiotic therapy whenever possible [166]. The use of beta-lactamase in dogs treated with ampicillin lowered its jejunal concentration and prevented it from reaching the colon [167]. This approach could lessen antibiotics' negative effects on the colonic microbial community. Ribaxamase (formerly SYN-004), an orally administered beta-lactamase with IV penicillins and cephalosporin, has shown promising results in preventing dysbiosis in hospitalized patients with lower respiratory tract infections treated with ceftriaxone [129]. A phase-2b study has shown a RCDI risk reduction in the interventional group [129]. This reduction occurred with macrolide plus ceftriaxone or ceftriaxone alone [129]. Ribaxamase is also effective when administered with beta-lactamase inhibitors like tazobactam and sulbactam [168]. Moreover, a phase-1b/2a clinical trial study to assess the safety, tolerability, and efficacy of orally administered SYN-004 in adult patients undergoing allogeneic hematopoietic cell transplantation is also ongoing [130].

### Adsorbent charcoal

The use of adsorbent charcoal is an additional approach to prevent antibiotic-induced dysbiosis. Dav-132 is a charcoal-based adsorbent that is actively studied [169]. It was developed for oncological patients for whom therapy is mandatory to prevent infections. Dav-132 is designed to become active in the ileum, cecum and colon before antibiotics could start impairing the GM [169, 170]. A phase-2 trial has confirmed that Dav-132 can be safely used in patients as it is well tolerated and promotes GM diversity by improving *Clostridioides difficile* colonization resistance [115]. Notably, a study on hamsters with moxifloxacin-induced *Clostridioides difficile* infection showed that a modified version of Dav-132 suitable for mice, namely Dav-131, could reduce mortality in a dose-dependent manner by lowering the fecal-free moxifloxacin concentration [114]. Therefore, despite lack of studies in critical illness, Dav-132 could potentially be an additional tool, together with probiotics administration, to reduce RCDI incidence and progression risks in ICU patients.

### Fecal microbiota transplantation

FMT is a medical technique in which stools from a "healthy" donor are delivered to a dysbiotic patient to restore eubiosis [2]. An FMT sample contains all microorganisms that naturally harbor in the gut and all their associated metabolites. For this reason, this treatment may restore dysbiosis better than others [171, 172]. However, this procedure does not come without risks. In fact, undesired and/or undetected pathogens could also be delivered from the donor to the recipient subject, sometimes even with fatal complications. In a case report, two patients developed drug-resistant *E. coli* bacteremia following FMT, causing one death [173]. Therefore, extreme caution should be taken, especially in critically ill patients with a higher risk of infection. In 2017, a panel of experts from the European Consensus Conference drafted FMT recommendations [132]. To date, the main indication for FMT is recurrent RCDI infection, where efficacy is superior to antibiotics [132, 174].

However, its application is currently being trialed in other conditions and could potentially be studied in critically ill patients [175], particularly to eradicate intestinal MDRO burden [176] and manage ICU common illnesses such as TBI [138], sepsis and multi-organ failure (MOF) [177].

A group of patients affected by RCDI with dysbiosis and severe gastrointestinal symptoms improved significantly after FMT, possibly via increased resistance MDROs’ gut colonization [176]. Intriguingly, FMT for sepsis has also been successfully used in patients with septic shock of undetermined cause with profuse diarrhea or diarrhea associated with antibiotic administration [139, 140]. FMT has been shown to promote eubiosis in a mouse model in which sepsis was induced post-administration of stool collected from a septic patient [177].

FMT has also been studied in TBI, which causes autonomic dysregulation, impaired BBB integrity, intestinal mucosal impairment, and brain immunity dysregulation. A recent study evaluated the effect of FMT following TBI in rats with a controlled cortical impact model, showing that this strategy could effectively restore eubiosis and resolve neurological deficits [138].

The emerging literature represents a great starting point for the exploitation of FMT in different ICU conditions and complications. Although European guidelines to regulate the use of FMT are in place, targeted RCTs should be initiated to explore the safety and efficacy of this procedure in the ICU without putting patients at risk of post-antibiotic discontinuation, a key step in FMT protocols. Rehorová et al*.* proposed an experimental standardized operating procedure for FMT in critically ill patients using a two-month-quarantined frozen multi-donor transplant administered by enema (seven donors, 50 ml from each donor to make a final graft of 350 ml). FMT should be done 48 h after the last antibiotic to maximize engraftment. Due to safety concerns, new studies should only include hemodynamically stable patients without perforated viscous or immunoparalysis [12].

## Conclusions

Recent evidence has shown that critically ill patients display a changed GM, known as pathobiota. The pathobiota composition is one of the leading causes of clinical complications. Metagenomic and meta-metabolomic studies are growing and dissecting mechanisms leading to dysbiosis in different ICU conditions. Harnessing the GM in ICU patients is intriguing, and this review summarizes the currently available results and outlines potential strategies for critically ill patients. Targeting intestinal bacteria has the potential to preserve or restore barrier integrity, which would then prime a beneficial cascade on the organ dysfunctions arising in ICU patients. New studies designed for critically ill patients should reinforce the current evidence, such as preventing VAP by probiotics, treating RCDI and MDRO colonization by FMT, preventing and resolving dysbiosis by personalized nutrition and antibiotic “damage control” tools, and have the potential to uncover critical biomarkers, stratify patients according to infection risk, immune response and inflammation status, and identify combination therapy/adjuvant responders vs. non-responders. Intensivists juggle the multiple connections between the intestine and the rest of the body. Unraveling this communication could transform them into modern "enterointensivists" at the front line of critical care management, placing the intestine at the center of critically ill patients as a "jack of all trades" card.

## Data Availability

Not applicable.

## Abbreviations

ALD: Alcoholic liver disease
AKI: Acute kidney injury
ARDS: Acute respiratory distress syndrome
BAs: Bile acids
BBB: Blood–brain barrier
CKD: Chronic kidney disease
COVID-19: The coronavirus disease 2019
EN: Enteral nutrition
ESRD: End-stage renal disease
FGF-19: Fibroblast growth factor 19
FMT: Fecal microbiota transplantation
FXR: Farnesoid X receptor
GBA: Gut–brain axis
GM: Gut microbiota
GF: Germ-free
HF: Heart failure
IBD: Intestinal bowel disease
ICU: Intensive care unit
IS: Indoxyl sulfate
LCFAs: Long-chain fatty acids
MDROs: Multidrug resistant bacteria
PCS: P-cresyl sulfate
PN: Parenteral nutrition
RCDI: Recurrent *clostridioides difficile *infection
SCFAs: Short-chain fatty acids
SOFAscore: Sequential organ failure assessment score
TBI: Traumatic brain injury
TMA: Trimethylamine
TMAO: TrimethylamineN­oxide
UT: Uremic toxins
VAP: Ventilator-associated pneumonia
